# The consequences of changes in exercise habits on work engagement and presenteeism: Evidence from an event-study analysis using Japanese longitudinal data

**DOI:** 10.5271/sjweh.4303

**Published:** 2026-09-01

**Authors:** Ryohei Kashima, Takuhiro Takada, Tomoaki Matsuo, Rina So

**Affiliations:** 1 National Institute of Occupational Safety and Health, Japan (JNIOSH).

**Keywords:** fixed effect, physical activity, quasi-experimental study

## Abstract

**Objectives:**

This study aimed to estimate the consequences of changes in employee exercise habits (starting and quitting) on work engagement and presenteeism.

**Methods:**

Since January 2023, the Japan Institute for Labour Policy and Training has conducted the semi-annual JILLS-i longitudinal, web-based survey to represent Japan’s middle-aged population structure (512 strata defined by gender, age, employment status, region, education). Of the 11 148 regular employees in the first wave, we analyzed the four-wave data of 6576 individuals (N=26 304 person-waves). We utilized work engagement and productivity (presenteeism) as our analytical outcomes. Binary indicators for relative time from changes in exercise habits were included as explanatory variables to estimate temporal associations with job-related outcomes using an event-study analysis, a quasi-experimental approach.

**Results:**

Both starting and quitting exercise habits showed no significant association with presenteeism. However, starting exercise was positively associated with work engagement, whereas quitting was negatively associated with it. Starting exercise showed a significant association over longer follow-up waves than quitting exercise. Gender differences were observed in the association of quitting exercise with work engagement.

**Conclusions:**

The acquisition of exercise habits may lead to a sustained improvement in work engagement, a crucial psychological resource for employees. Our results potentially suggest a divergence in the dynamics of effect between organizational support for starting versus preventing quitting exercise. Although exercise promotion may not lead to substantial improvements in productivity as measured by presenteeism, it may still yield meaningful psychological benefits.

Employee health and motivation are crucial for both individual well-being and organizational sustainability. Poor health is linked to absenteeism (not attending scheduled work due to sickness) and presenteeism (attending work while ill) (1, 2). From an organizational productivity perspective, absenteeism results in zero production output, whereas presenteeism involves some production but represents a state of reduced productivity compared with employees in good health (2). Because presenteeism often incurs higher economic costs than absenteeism due to its frequency (3, 4), it has become an important concern for managers. Concurrently, organizations are increasingly prioritizing work engagement, a positive and fulfilling psychological state that is associated with superior job performance and low turnover (5–8). Consequently, finding effective strategies to enhance work engagement while mitigating presenteeism is an increasing priority for occupational health.

Many organizations have implemented wellness programs that are expected to improve employee health and well-being, reduce presenteeism, and enhance work engagement (9). Since the physical and mental health benefits of exercise are well established (10, 11), promoting exercise habits is a central strategy for wellness programs (9). Despite widespread awareness of exercise benefits, lifestyle-related diseases such as obesity remain significant public health issues (12, 13). Reliance solely on individual willpower to maintain healthy behaviors has clear limitations (14); therefore, organizational support can help employees overcome these challenges.

Exercise habits are positively associated with work engagement, negatively associated with burnout (15, 16), and negatively associated with presenteeism (2, 17). Because regular exercise is associated with better physical and mental health (10, 11), it may be associated with a lower likelihood of attending work while ill and with less impaired work functioning (2). In addition, successfully maintaining an exercise habit may enhance self-efficacy and positive affect, which could in turn relate to higher work engagement. However, most existing research relies on cross-sectional designs, which faced the following problems (2, 17). First, associations may be biased by reverse causality, where highly productive or engaged individuals are more likely to exercise (2). Second, unobserved confounders, such as stable personality traits, may simultaneously influence both exercise behavior and psychological outcomes (18). To address these limitations, recent studies accumulate results from randomized controlled trials in exercise-based workplace wellness programs (19–22) and from quasi-experimental analyses (23–25), but further studies are essential.

In the context of exercise effects, the primary interest lies in the benefits of starting exercise. While some studies examined whether exercise can be maintained as an outcome (19, 21), little attention has been paid to the adverse effects of quitting exercise. Due to the dynamic nature of exercise habits, individuals fluctuate between starting, maintaining, and quitting habits. Regression analyses that rely on binary indicators of exercise habits implicitly assume symmetric associations, meaning the benefits of starting exercise are equal in magnitude to the drawbacks of quitting. However, Prospect Theory demonstrates that gains and losses often have asymmetric psychological impacts (26). In the context of exercise habits, no evidence currently confirms whether starting and quitting exercise habits exert symmetric or asymmetric effects. As an adverse event, quitting exercise may trigger adaptive responses similar to the “Psychological Immune System” to mitigate distress (27). Conversely, the acquisition of regular exercise, a difficult-to-maintain habit, could produce strong positive effects mediated by psychological constructs such as self-efficacy (28). If an effect discrepancy exists, regression coefficients calculated as weighted averages may misinterpret the associations of starting and quitting exercise habits. To understand how changes in exercise habits influence work engagement and presenteeism, it is necessary to distinguish between starting and quitting and to examine how their associations evolve over time.

This study aimed to utilize large-scale longitudinal panel data from Japan to estimate the asymmetric and time-varying associations of starting and quitting exercise with work engagement and presenteeism. Using a quasi-experimental “event-study” approach that controls for individual fixed-effects, we aim to clarify these associations close to causality. Furthermore, considering documented gender differences in health behaviors (29), we examine whether these associations vary between male and female employees.

## Methods

We secondarily utilized data from JILLS-i, the web-based, semi-annual Japan Institute for Labour Policy and Training (JILPT) longitudinal survey of middle-aged individuals (aged 35–54 years at baseline) (30). The survey employed a quota sampling design to ensure representativeness of the population based on Japan’s 2020 national census. Quotas for the target sample of 20 000 respondents were established across 512 strata, defined by cross-classifications of five variables: gender (male/female), age group (5-year increments), employment status (regular/non-regular/self-employed/unemployed), residential area (eight regions), and education level (university/non-university) (30). Data were collected across four waves between January 2023 and July 2024.

The JILPT Ethical Committee approved the survey (29 November 2022, No. R4-01) and secondary analyses, including this study (25 March 2025, JILPT No. 2). All participants provided informed consent electronically prior to completing each survey.

### Analytical sample

The baseline survey (wave 1) included 11 148 regular employees. For the analytical sample, we used complete-case analysis, including only participants with data available at all four waves to handle missing repeated answers. We restricted the sample to individuals who maintained regular employment across all waves to minimize confounding factors from employment changes, which could affect leisure time, income, and exercise habits. Finally, we analyzed complete longitudinal panel data from 6576 individuals, contributing a total of 26 304 person-wave observations.

### Data

*Exercise habits.* Exercise habits were assessed using a single question: “Do you exercise for at least 30 minutes twice a week or more, enough to make you sweat lightly?” (yes=1; no=0). Our question focused solely on the presence or absence of exercise habits. Simple physical activity questions have been discussed in population surveillance research as pragmatic tools for capturing broad behavioral differences (31). Additionally, a Japanese study using a similar questionnaire found that exercise-habit status was positively associated with cardiorespiratory fitness, suggesting that brief measures capture key differences in habitual exercise (32). For the primary analysis, we employed binary indicator variables corresponding to the relative wave from the baseline wave when individuals initially changed their exercise habits (ie, started or quit). Relative wave -2 indicates two waves prior to the change, relative wave 0 indicates the period immediately after the change, and relative wave 2 indicates the period two waves after the change in exercise habits. One wave corresponds to 0.5 years.

*Work engagement*. Work engagement is a positive, fulfilling, work-related state characterized by vigor, dedication, and absorption (6, 7). The three-item Utrecht Work Engagement Scale was used (6, 7), with respondents answering on a 7-point scale ranging from 1=not at all to 7=always (every day). The mean score of the three items was used for analysis, after subtracting 1 from each item value to align with the original scale, meaning the higher the score, the higher the level of engagement. The reliability and validity of the Japanese version have also been confirmed (7).

*Presenteeism.* The Single-Item Presenteeism Question (SPQ) was administered, which asks for a response of 1–100%^1^, indicating one’s work performance over the past four weeks compared to healthy conditions (33). The response value was used to measure presenteeism (SPQ score=100% - response value) and is almost equivalent in constructive validity and responsiveness to the WHO’s Health and Work Performance Questionnaire (33, 34). The SPQ is a questionnaire originally developed in Japan. We multiplied the SPQ scores by 1/10 for easy interpretation since multiplying by a scalar does not affect the statistical significance of the results. The higher the value, the more productivity was impaired.

### Covariates

Main analyses included only time (survey wave) and individual fixed effects, controlling for numerous time- and individual-specific factors, including unobservable factors (35). To address remaining potential confounding from time–individual interactions (eg, birth of a child, marriage, changes in working hours), we conducted sensitivity analyses controlling for weekly working hours and dummy variables for marital status, presence of children, and life events in the past 6 months (marriage, birth of a child, child advancement in school level, divorce, spouse changing job, spouse quitting job, severe family illness, family requirement for caregiving). Self-reported gender, age, firm size, and industry were reported descriptively but not included in main analyses, as they are absorbed by individual fixed-effects.

### Statistical analysis

Our data collected exercise habits status across four waves, containing a total of 2^4^ patterns. To simplify interpretation, we classified individuals with temporal exercise habit patterns into the following five types: (i) never engaged in exercise habits (“never exercise”); (ii) started and subsequently maintained (“exercise starter”); (iii) experienced ≥2 changes in exercise habits (“exercise switcher”); (iv) quit and subsequently maintained (“exercise quitter”); and (v) always engaged in exercise habits (“always exercise”).

To distinguish the association of starting exercise habits from that of quitting, we conducted distinct subsample analyses rather than pooling participants. For starting exercise, we compared type “exercise starter” with “never exercise.” For quitting exercise, we compared “exercise quitter” with “always exercise”. This approach avoids assuming symmetric association size between starting and quitting exercise (33–35).

Recent econometric studies recommend avoiding cases in which the treatment status is reversed and the sample simultaneously contains both directional treatments (36–38). Additionally, conventional regression analyses on the binary exercise indicator with the entire sample implicitly assume that the benefits of starting the habit and the disadvantages of quitting are symmetrical. However, the effects thereof may differ. Our subsample analysis can avoid these issues.

We estimated dynamic associations of newly starting or quitting exercise habits on job-related outcomes using an event-study approach with a two-way fixed-effects model (39), which accounts for unobserved wave- and individual-specific confounders. The coefficient of binary indicators for relative waves represent the association on each relative period, which evolves over time. Cluster-robust standard errors were calculated at the individual level (35). Statistical significance was set at P<0.05.

As a sensitivity analysis, we conducted separate analyses for male and female subsamples. Work engagement and presenteeism are subjective outcomes, and responses may differ between genders (40). Furthermore, to confirm the robustness of the results, we performed an estimate with a robust to differences in timing of changes in exercise habits (41–43), and an estimate controlling for working hours, family situations, and life events, which may influence the presence of exercise habits.

Statistical analysis was performed using STATA 17 MP (StataCorp LLC, College Station, TX, USA) with the “reghdfe” and “jwdid” commands. A detailed flowchart of the participant and sample selection process is provided in supplementary material, www.sjweh.fi/article/4303, figure S1. Details of the analysis are described in supplementary appendix A.

## Results

Table 1 presents descriptive statistics of the five types of exercise habit patterns over time: “never exercise” (53.5% of the cohort), “always exercise” (17.8%), “exercise switcher” (14.2%), “exercise starter” (9.3%), and “exercise quitter” (5.2%).

**Table 1:** Four-wave pooled descriptive statistics by five types of exercise habits ^a^ patterns over time. [SD=standard deviation]

		Combined all (N=26 304)					Exercise habit starting effect analytical samples										Non-analytical samples					Exercise habits quitting effect analytical samples								
							Never exercise					Exercise starter					Exercise switcher					Exercise quitter					Always exercise			
							(N=14 076, 53.5%)					(N=2436, 9.3%)					(N=3732,14.2%)					(N=1376, 5.2%)					(N=4684, 17.8%)			
		N	%	Mean	SD		N	%	Mean	SD		N	%	Mean	SD		N	%	Mean	SD		N	%	Mean	SD		N	%	Mean	SD
Work engagement				2.45	1.47				2.29	1.45				2.58	1.48				2.59	1.41				2.52	1.51				2.74	1.47
Presenteeism				2.42	2.33				2.52	2.36				2.29	2.32				2.47	2.31				2.42	2.36				2.14	2.21
Gender																														
	Male	18 656	70.9				9112	64.7				1784	73.2				2868	76.8				1064	77.3				3828	81.7		
	Female	7648	29.1				4964	35.3				652	26.8				864	23.2				312	22.7				856	18.3		
	Age			46.07	5.39				46.27	5.36				45.69	5.42				45.68	5.40				45.97	5.57				46.03	5.40
	Education years			14.20	1.99				14.12	1.98				14.19	1.92				14.22	1.99				14.22	2.06				14.43	2.01
Industry																														
	Primary	184	0.7				83	0.6				35	1.4				35	0.9				5	0.4				26	0.6		
	Manufacture	7672	29.2				4099	29.1				789	32.4				1062	28.5				385	28.0				1337	28.5		
	Service	16 175	61.5				8930	63.4				1349	55.4				2263	60.6				840	61.0				2793	59.6		
	Public & Other	2273	8.6				964	6.8				263	10.8				372	10.0				146	10.6				528	11.3		
Firm Size																														
	1–49	6189	23.5				3602	25.6				566	23.2				887	23.8				233	16.9				901	19.2		
	50–299	6558	24.9				3520	25.0				630	25.9				868	23.3				375	27.3				1165	24.9		
	>300	9908	37.7				5085	36.1				880	36.1				1443	38.7				556	40.4				1944	41.5		
	Unknown	3649	13.9				1869	13.3				360	14.8				534	14.3				212	15.4				674	14.4		
Working hours				37.36	16.61				37.67	16.44				36.63	16.91				36.37	17.05				37.58	17.00				37.53	16.43
Married																														
	Yes	14 673	55.8				7689	54.6				1409	57.8				2181	58.4				759	55.2				2635	56.3		
	No	11 631	44.2				6387	45.4				1027	42.2				1551	41.6				617	44.8				2049	43.7		
Children																														
	Yes	13 318	50.6				6981	49.6				1262	51.8				1981	53.1				689	50.1				2405	51.3		
	No	12 986	49.4				7095	50.4				1174	48.2				1751	46.9				687	49.9				2279	48.7		
Marriage		144	0.5				51	0.4				13	0.5				33	0.9				6	0.4				41	0.9		
Birth of a child		206	0.8				91	0.6				20	0.8				38	1.0				10	0.7				47	1.0		
Child’s entrance exams		1768	6.7				878	6.2				162	6.7				264	7.1				98	7.1				366	7.8		
Divorce		136	0.5				72	0.5				11	0.5				27	0.7				7	0.5				19	0.4		
Spouse’s job status change		455	1.7				218	1.5				47	1.9				78	2.1				23	1.7				89	1.9		
Spouse’s unemployment		470	1.8				227	1.6				40	1.6				96	2.6				22	1.6				85	1.8		
Family illness		1264	4.8				681	4.8				93	3.8				195	5.2				73	5.3				222	4.7		
Family caregiving		986	3.7				572	4.1				61	2.5				150	4.0				46	3.3				157	3.4		

^a^ % of exercise habits in all four waves sample is 30.9%. Detail exercise status changes over time are demonstrated in supplementary table S1.

In the pooled sample, 29% of the respondents were female, with a mean age of 46 years; 56% of the respondents had a spouse. The most common industry was the service industry (61%), and the most common company size was >300 employees (38%).

We recorded 31% of respondents as having exercise habits, which is close share in previous Japanese study (32). “Never exercise” had the worst work engagement and presenteeism, and “always exercise” had the best situation in both outcomes. Supplementary table S1 details the patterns of exercise habits over time.

### Dynamic association of changes in exercise habits

**Figure 1:**
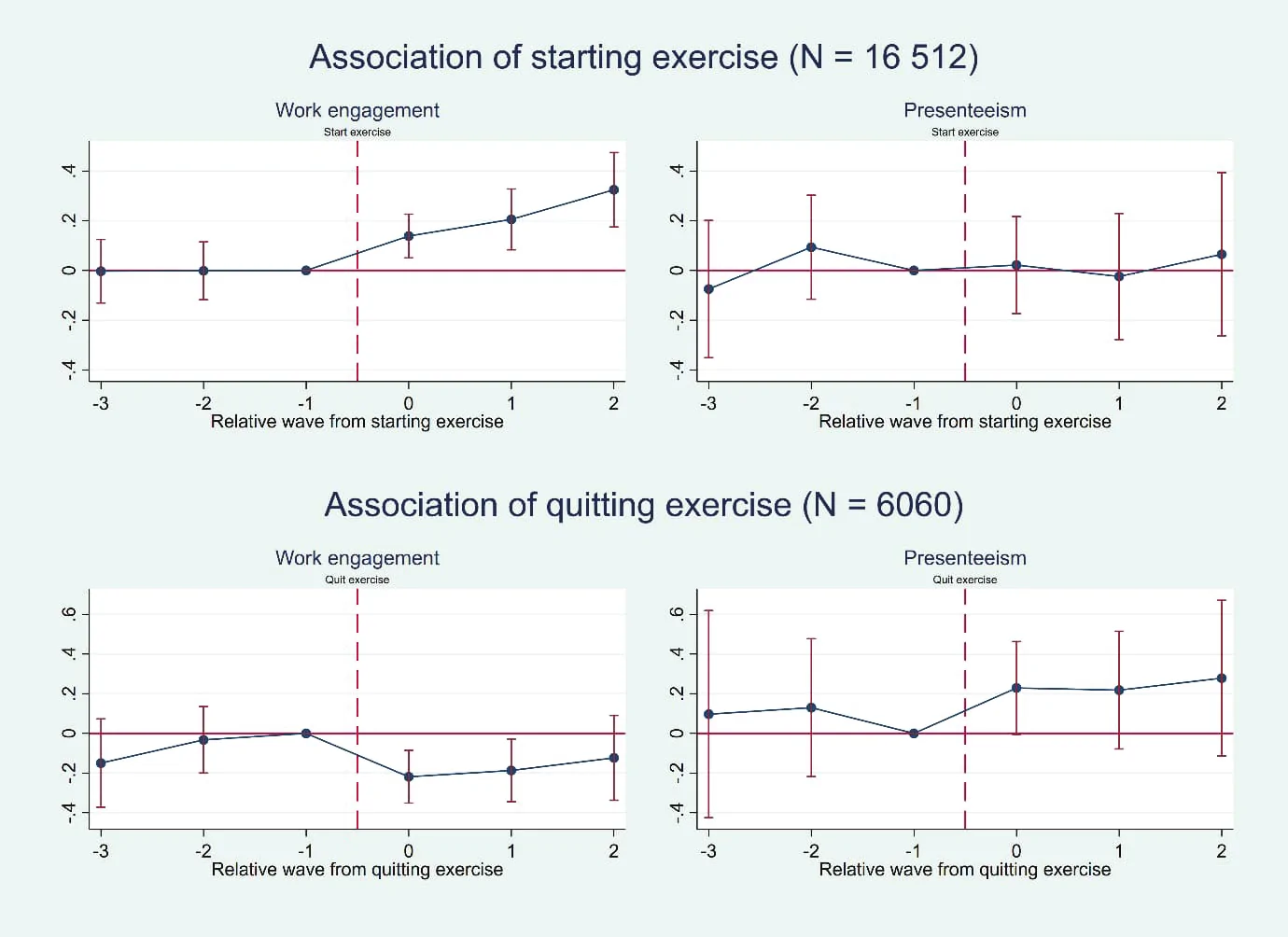
Association dynamics of changes in exercise habits on work engagement and presenteeism. **Note:** The upper part of the figure estimates the association for starting exercise habit with work engagement and presenteeism by using a subsample of only the “never exercise” and “always exercise”groups (N=16 512). In contrast, the lower part estimates the association for quitting exercise habits by using a subsample of only the “exercise starter” and “exercise quitter” groups (N=6060). The x-axis shows the relative wave from the survey wave where exercise habits changed. The area to the left of the vertical dashed line indicates the association before the change in exercise habits, while the area to the right indicates the association after the change in exercise habits. The dots indicate the point estimates. The lines surrounding the dots indicate the 95% confidence interval. Supplementary table S2. coincides with this figure. Details of regression appear in appendix A.

Figure 1 shows point estimates and 95% confidence intervals (CI) for the associations of exercise habit changes (starting or quitting) with work engagement and presenteeism across relative waves. The upper panels depict coefficients for starting exercise, while the bottom panels show coefficients for quitting. “Exercise switcher” was not included in the dynamic association analyses due to estimation limitations.

Focusing on starting exercise, no significant coefficients were observed for either outcome in the periods before newly starting exercise habits. For work engagement, positive coefficients were observed after starting exercise, with the absolute value of point estimation gradually increasing over time relative to each wave (wave 0: β=0.138, 95% CI 0.050–0.226, P=0.002; wave 1: β=0.205, 95% CI 0.083–0.328, P=0.001; wave 2: β=0.324, 95% CI 0.175–0.474, P<0.001). For presenteeism, no significant coefficients were observed after starting exercise, and point estimates remained stable at approximately 0 (wave 0: β=0.022, 95% CI -0.174– 0.217, P=0.828; wave 1: β= -0.024, 95% CI -0.278–0.229, P=0.852; wave 2: β=0.065, 95% CI -0.263–0.393, P=0.697).

Next, focusing on quitting exercise, we confirmed that no significant coefficients were observed for either outcome in the prior period. For work engagement, significant negative coefficients were observed immediately after quitting exercise and at the next wave. However, no statistically significant effect was demonstrated by the second wave (wave 0: β= -0.219, 95% CI -0.352− -0.086, P=0.001; wave 1: β= -0.187, 95% CI -0.344− -0.030, P=0.020; wave 2: β= -0.123, 95% CI -0.338–0.091, P=0.258). The estimated coefficients for each period showed a gradual reduction in absolute value over time. Regarding presenteeism, although the point estimates showed stable positive coefficients after quitting exercise, no significant coefficients were observed (wave 0: β=0.229, 95% CI -0.005–0.463, P=0.055; β=0.218, 95% CI -0.079–0.515, P=0.150; β=0.278, 95% CI -0.114–0.671, P=0.165). Supplementary table S2 details the point-estimation results in figure 1.

### Heterogeneities between males and females

Since work engagement and presenteeism are subjective outcome variables, potential gender differences in perception may moderate the associations of changes in exercise habits. We conducted a gender-stratified subsample analysis to estimate the associations of starting and quitting exercise, with the same format as figure 1.

**Figure 2:**
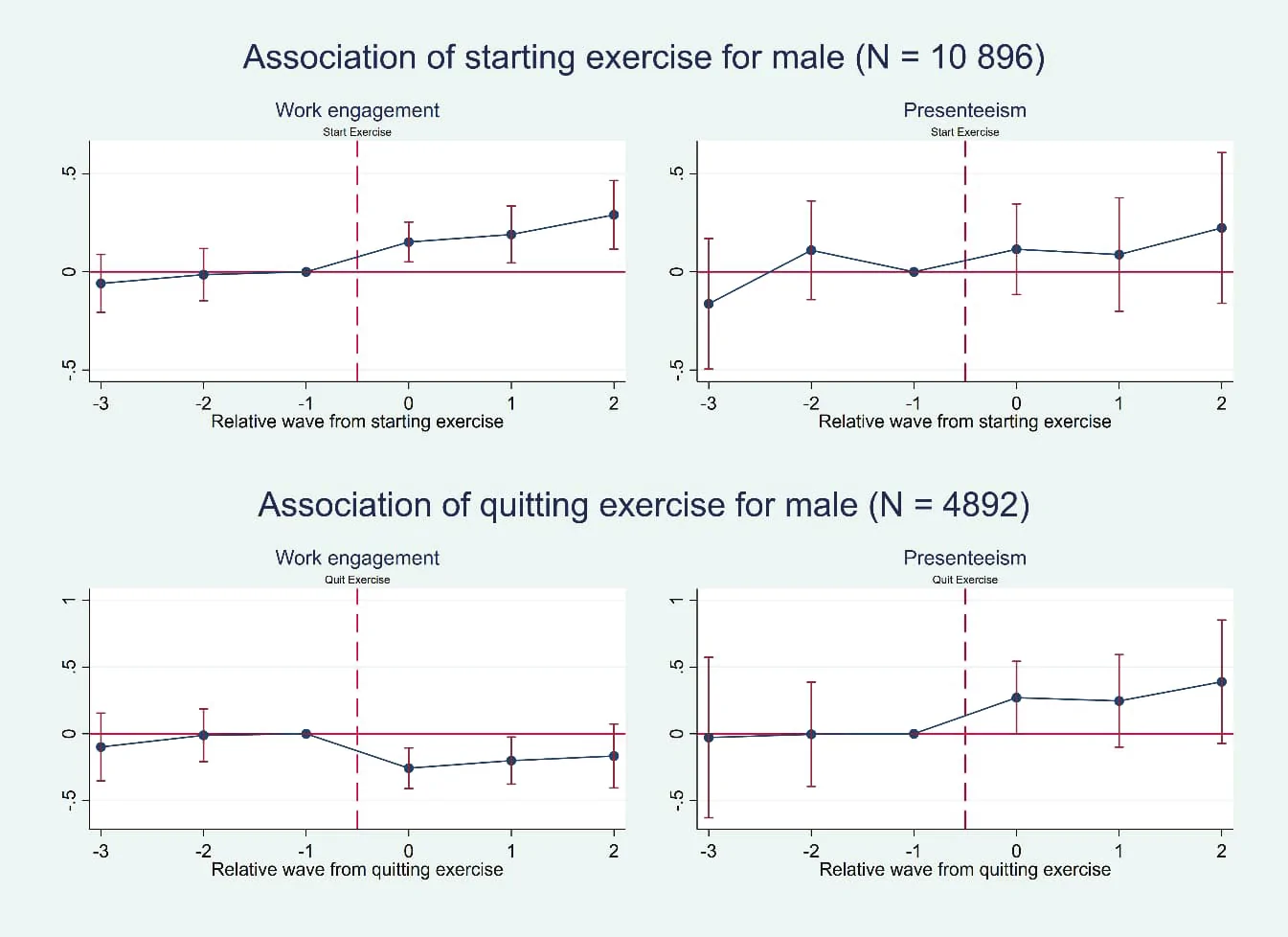
Association dynamics of changes in exercise habits on work engagement and presenteeism for male subsample. **Note:** The upper part of the figure estimates the association for starting exercise habit with work engagement and presenteeism by using a male subsample of only “never exercise” and “always exercise”groups (N=10 896). In contrast, the lower part estimates the association for quitting exercise habits by using a male subsample of only “exercise starter” and “exercise quitter” groups (N=4892). The x-axis shows the relative wave from the survey wave where exercise habits changed. The area to the left of the vertical dashed line indicates the association before the change in exercise habits, while the area to the right indicates the association after the change in exercise habits. The dots indicate the point estimates. The lines surrounding the dots indicate the 95% confidence interval.Supplementary table S3. coincides with this figure. Details of regression appear in appendix A.

Figure 2 presents the results for males. As males constituted most of our sample, these findings are consistent with the full sample results presented in figure 1. Consistent with the full sample, starting exercise was associated with sustained significant increases in work engagement, while no significant associations were observed for presenteeism. However, the later dynamics did not fully align. In the full sample, the dynamics stabilized at approximately 0, whereas for the male sample, it stabilized at positive values. For quitting exercise, we confirmed the same statistical significances and dynamics of point estimations for both outcomes as shown in figure 1.

**Figure 3:**
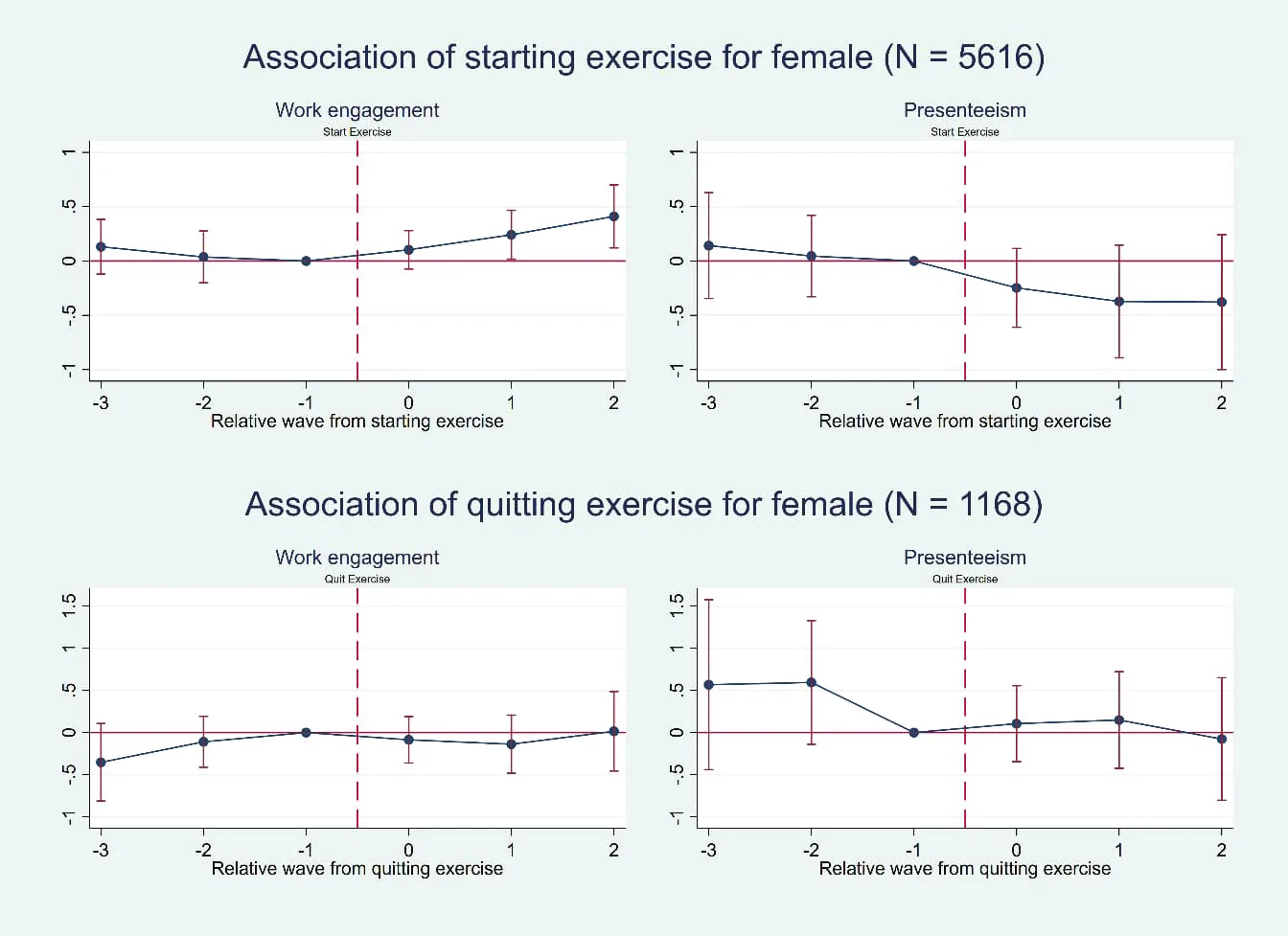
Association dynamics of changes in exercise habits on work engagement and presenteeism. **Note:** The upper part of the figure estimates the association for starting exercise habit with work engagement and presenteeism by using a female subsample of only “never exercise” and “always exercise”groups (N=5616). In contrast, the lower part estimates the association for quitting exercise habits by using a female subsample of only “exercise starter” and “exercise quitter” groups (N=1168). The x-axis shows the relative wave from the survey wave where exercise habits changed. The area to the left of the vertical dashed line indicates the association before the change in exercise habits, while the area to the right indicates the association after the change in exercise habits. The dots indicate the point estimates. The lines surrounding the dots indicate the 95% confidence interval. Supplementary table S3. coincides with this figure. Details of regression appear in appendix A.

Figure 3 displays the results for females. For work engagement, although starting exercise shows a similar pattern to positive dynamic coefficients as in males, quitting exercise shows stable coefficients near 0 and no significances, which differ from the results in males. For presenteeism, the lack of significances across all periods in females were also consistent with the findings presented in males. However, the shapes of dynamics of point estimations differed. For presenteeism, starting exercise habits in females showed negative coefficients and an increasing trend in absolute values, whereas in males, coefficients are stable and positive. Quitting exercise habits in females shows stable coefficients near 0, whereas in males, they show positive coefficients. Supplementary table S3 details the estimation results for figures 2 and 3.

### Sensitivity analysis

Supplementary table S4 and figure S2 present robustness estimates accounting for heterogeneity in the timing of exercise habits changes. Supplementary table S5 and figure S3 show the results of the event-study estimation additionally controlling for working hours, marital status, presence of children, and life event indicators. These results support the robustness of the dynamic patterns observed in figure 1.

## Discussion

### Summary of results

Our study employed quasi-experimental methods using four-wave longitudinal data from regular employees in Japan to examine the dynamic associations of starting and quitting exercise habits with work engagement and presenteeism. Key findings are that (i) there is an asymmetry of duration of associations between starting and quitting exercise habits on work engagement; (ii) changes in exercise habits (both starting and quitting) have no statistically significant associations with presenteeism; and (iii) the negative association of quitting exercise habits with work engagement is significant only among males.

### Asymmetry of the dynamic association between starting and quitting exercise habits on work engagement

We observed asymmetry in the coefficients of starting and quitting exercise habits on work engagement. The positive coefficients for starting exercise remained significant at 1 year (relative wave 2) after starting. In contrast, the negative significant coefficients of quitting continued up to 0.5 years (relative wave 1) after quitting, and disappeared 1 year later (relative wave 2). Our work engagement results indicate a positive relationship between exercise habits and work engagement, consistent with previous research (22, 44, 45).

As a potential psychological mechanism behind this asymmetry, starting exercise habits may have led to an increase in self-efficacy (28). The experience of acquiring exercise habits, a difficult habit to maintain, enhances an individual’s self-efficacy. This heightened self-efficacy could induce a spillover effect on the work domain, increasing persistence and motivation toward challenging tasks, leading to a sustained improvement in work engagement (46–48).

Conversely, the temporary nature of the negative associations for quitting exercise with work engagement may be explained by Adaptation Level Theory (49). Abruptly quitting exercise was negatively associated with work engagement. However, workers eventually adapt to the absence of exercise, and work engagement returns to its original level. This finding is consistent with prior research showing that while work engagement is a stable trait over the long term, it is also influenced by short-term fluctuating factors (50).

### No significant association with presenteeism

In contrast to work engagement results, neither starting nor quitting exercise significantly affected presenteeism. Our result aligns with previous studies that found no significant positive effect of organizations’ exercise support type wellness programs on job performance (19, 20, 22), but contradicts others reporting a positive significant effect (23, 51).

Our results may stem from the complexity of presenteeism. Presenteeism involves a complex interplay of numerous factors, including high job demands, organizational cultures that heighten fear of dismissal, organizational and social factors such as perceived obligations toward colleagues, and specific health issues like musculoskeletal pain and mental distress (2). Even if exercise habits cultivate psychological resources like self-efficacy, this may be insufficient to improve health status or productivity performance in the short term. In other words, benefits of exercise habits may be limited to specific domains in the short term. While exercise habits influence emotional and energetic aspects, such as work engagement, exercise can have no direct effect on enhancing actual job performance, as measured by presenteeism.

While the associations for changes in exercise habits with presenteeism were not statistically significant, some coefficients were stable in the post-waves: for men, quitting; for women, starting. We must recognize that as the sample size increases, the existence of these effects could be supported.

### Similarity and difference from the results of Dutch lifestyle intervention RCT

Our findings, no significant associations with presenteeism and a significant association with work engagement, align closely with the Dutch lifestyle intervention randomized controlled trial (RCT) (22). Their program showed no significant effects on productivity (presenteeism) or absenteeism, but demonstrated significant effects on psychological outcomes, such as vitality, among high-frequency participants.

Despite similar results, the analytical focus of the two studies differs. Strijk et al (22) estimated intention-to-treat effects of an assigned intervention, emphasizing the average program effect, whereas our quasi-experimental design focused on associations with realized changes in individual exercise habits. Importantly, the high-frequency participants who showed favorable effects in Strijk et al (22) may resemble the sustained exercise starters identified in our study, which could partly explain the convergence in findings. While the RCT offers high internal validity for causal inference, its sample size (N=730) and composition, which consists mostly of relatively older female hospital workers, limit generalizability (22). In contrast, although our study is weaker in terms of causal interpretation compared with an RCT, its larger and more diverse sample may provide greater external validity.

Viewed together, the two studies complement each other and suggest that the conclusion drawn by Strijk et al (22) – that exercise interventions are more likely to enhance engagement or vitality through sustained behavioral change rather than immediate productivity gains – is likely to be broadly generalizable. Another notable difference in our research was that, by focusing on the dynamic nature of exercise, we gained further insights into the negative consequences of quitting exercise.

### Asymmetrical association between genders

Another notable finding is the gender asymmetry of quitting exercise habits. The temporally significant negative association with work engagement was observed only among male and not female employees. While we cannot directly identify the mechanisms underlying this difference, several possibilities can be considered. One possible explanation involves gender differences in stress coping strategies (52). Male employees may rely more heavily on exercise habits as a primary stress reliever and source of psychological energy; when deprived of it, they might struggle to find immediate alternatives. In contrast, female employees may possess a more diverse portfolio of stress coping strategies, allowing them to compensate with other resources after quitting exercise. Additionally, the reasons for quitting exercise and how the free time is used afterward may differ between genders. One possibility is that male workers began working longer hours after quitting exercise habits. Supplementary figure S4 and table S6 confirm that neither men nor women increased their working hours after quitting exercise habits, using the same analysis as in figure 1 (outcome=working hours). Thus, the gender difference stems from changes other than working hours after quitting exercise. However, our study could not fully unpack this mechanism.

### Implications for organizational wellness practice

Our findings have several implications for organizational wellness practices to promote exercise habits. First, our results for work engagement suggest a divergence in the dynamics of effect between organizational support for starting and preventing quitting. Figure 1 suggested that the positive association from starting exercise lasted longer than the negative association from quitting. If the associations we have demonstrated were a causal effect, it is conceivable that the impact of organizational support may differ depending on whether it focuses on encouraging employees to start exercise or on preventing them from quitting exercise. One possibility is that focusing resource allocation on reducing barriers to exercise participation could be more efficient. Second, our findings on gender asymmetry in quitting exercise suggest that the effectiveness of organizational support in preventing employees from quitting exercise may be limited when the proportion of female employees is higher, suggesting limitations to a one-size-fits-all approach. Third, our results reinforce the view drawn by Strijk et al’s RCT (22) that exercise interventions are more likely to yield psychological returns rather than immediate productivity gains, suggesting that occupational health and safety staff planning exercise interventions should set realistic outcomes as their goals.

From a perspective that goes beyond the organization’s wellness initiatives, adjustment of job demand and control allocation may facilitate employees’ recovery from work and encourage exercise after work (53), which potentially links to the positive association between exercise and work engagement observed in our study. Therefore, these findings imply that organizational support for employee health may need to extend beyond conventional wellness programs and include work design features that create room for recovery and exercise.

### Strength and limitations

A primary strength of this study is the use of a quasi-experimental event-study design to examine the temporal associations between changes in exercise habits (both starting and quitting) and work outcomes (engagement and presenteeism). Importantly, the absence of significant coefficients prior to the habit changes indicates that the ex-ante common trends assumption was not violated, which supports the validity of an interpretation close to a causal relationship for our association results.

However, this study has several limitations. First, exercise habits were assessed using a single self-reported questionnaire item, without formal validation against established physical activity measures. Objective indicators such as accelerometer data or detailed information on exercise frequency, intensity, type, duration, or context were not collected. We also did not assess whether exercise was initiated or supported by the workplace or whether it occurred during paid work hours. Therefore, our findings should be interpreted as reflecting broadly defined individual exercise habits, rather than structured workplace exercise programs. Second, while this study demonstrated the significant association between exercise and work engagement, it did not directly examine the underlying psychological mechanisms (ie, self-efficacy, stress reduction) or factors contributing to gender differences. Elucidating these mechanisms requires an approach that combines mediation analysis with qualitative research. Third, as the analysis was limited to regular employees in Japan, caution is needed when generalizing to non-regular employees or different cultural contexts. Our sample restriction may lead to a conservative estimation about quitting exercise, especially if regular employees transition to unemployment due to deteriorating health. Fourth, further examination is needed regarding the heterogeneity of associations beyond gender differences. Fifth, the point estimation at the endpoint of the analysis period in the event study (eg, relative time point 2) was estimated from only the group changed habits at wave 2, resulting in lower results’ robustness than the case immediately after habit change (relative period 0). Sixth, the “exercise switcher” group which accounted for 14.2% of the sample, was excluded from the analysis due to the approach. Therefore, generalizability is limited. However, we do not support the claim that including the group is better than excluding it for estimating association dynamics of starting and quitting exercise from a perspective close to a causal relationship. Finally, although we emphasize implications for organizational wellness practices, the effect of company-level interventions could differ from those observed in this individual-level study.

Despite these limitations, our findings provide valuable insights into the divergent associations between starting and quitting exercise habits and occupational outcomes. This is particularly relevant for quitting exercise, as conducting an RCT is ethically challenging, making observational studies such as ours especially valuable for understanding these dynamics.

### Concluding remarks

Our results suggest a divergence in the dynamics of association between organizational support for starting and the mitigation of quitting exercise. Our findings suggest that organizations should not highly expect positive effects on productivity (presenteeism) driven by exercise promotion, but should anticipate psychological return. Neither starting nor quitting exercise has a significant impact on presenteeism. Future research should uncover the mechanisms underlying asymmetric associations in work engagements and verify whether asymmetry persists over longer periods or is observable across different cultural and occupational contexts.

## Supplementary material

Supplementary file 1

## Data Availability

This paper utilizes microdata from JILPT, and we do not have the authority to share the data. Our data can be accessed after reasonable requests to JILPT (www.jil.go.jp/english/index.html). We can offer the STATA command file as requested.
