# Supplementary material for: The consequences of changes in exercise habits on work engagement and presenteeism: Evidence from an event-study analysis using Japanese longitudinal data
Source: Scand J Work Environ Health. 2026 Aug 31;52(5):570–81. doi: 10.5271/sjweh.4303 (PMC13551495; doi:10.5271/sjweh.4303)
Supplement: Supplementary file 1 [file SJWEH-52-570-S001.pdf]

The consequences of changes in exercise habits on work engagement and presenteeism: Evidence from an event-study analysis using Japanese longitudinal data[1]

by Ryohei Kashima, PhD,[2] Takuhiro Takada, PhD, Tomoaki Matsuo, PhD, Rina So, PhD

1. Supplementary material
2. Correspondence to: Ryohei Kashima, National Institute of Occupational Safety and Health, 214-8585 6-21-1 Nagao, Tama-ku, Kawasaki city, Kanagawa prefecture, Japan. [E-mail: kashima-r@h.jniosh.johas.go.jp]

Table S1. Detailed 2^4 patterns of exercise status changes over time

		5 type exercise patterns over time						
		(A) Never Exercise	(B) Exercise Starter	(C) Exercise Switcher	(D) Exercise Quitter	(E) Always Exercise	Total	%
Detailed exercise pattern over time ("abcd") a: wave4 b: wave3 c: wave2 d: wave1	0000	3519	0	0	0	0	3519	53.5%
	0001	0	0	0	162	0	162	2.5%
	0010	0	0	314	0	0	314	4.8%
	0011	0	0	0	89	0	89	1.4%
	0100	0	0	105	0	0	105	1.6%
	0101	0	0	25	0	0	25	0.4%
	0110	0	0	55	0	0	55	0.8%
	0111	0	0	0	93	0	93	1.4%
	1000	0	265	0	0	0	265	4.0%
	1001	0	0	53	0	0	53	0.8%
	1010	0	0	195	0	0	195	3.0%
	1011	0	0	131	0	0	131	2.0%
	1100	0	102	0	0	0	102	1.6%
	1101	0	0	55	0	0	55	0.8%
	1110	0	242	0	0	0	242	3.7%
	1111	0	0	0	0	1171	1171	17.8%
	Total	3519	609	933	344	1171	6576	100%
	%	53.5%	9.3%	14.2%	5.2%	17.8%	100%	

Table S2. Association dynamics of changes in exercise habits on work engagement and presenteeism

		Association of starting exercise (N=16 512)				Association of quitting exercise (N=6060)			
Outcome	Relative wave	Coefficient	[95%CI]	(SE)	p-value	Coefficient	[95%CI]	(SE)	p-value
Work engagement	Lead3	-0.003	[-0.131,0.125]	(0.065)	0.965	-0.150	[-0.373,0.073]	(0.114)	0.187
	Lead2	-0.002	[-0.118,0.115]	(0.059)	0.979	-0.033	[-0.200,0.135]	(0.085)	0.701
	Lead1	Base	Base	Base	Base	Base	Base	Base	Base
	Lag0	0.138**	[0.050,0.226]	(0.045)	0.002	-0.219**	[-0.352,-0.086]	(0.068)	0.001
	Lag1	0.205***	[0.083,0.328]	(0.062)	0.001	-0.187*	[-0.344,-0.030]	(0.080)	0.020
	Lag2	0.324***	[0.175,0.474]	(0.076)	<0.001	-0.123	[-0.338,0.091]	(0.109)	0.258
Presenteeism	Lead3	-0.075	[-0.351,0.201]	(0.141)	0.596	0.097	[-0.426,0.620]	(0.266)	0.716
	Lead2	0.094	[-0.115,0.303]	(0.107)	0.380	0.130	[-0.218,0.478]	(0.177)	0.465
	Lead1	Base	Base	Base	Base	Base	Base	Base	Base
	Lag0	0.022	[-0.174,0.217]	(0.100)	0.828	0.229	[-0.005,0.463]	(0.119)	0.055
	Lag1	-0.024	[-0.278,0.229]	(0.129)	0.852	0.218	[-0.079,0.515]	(0.151)	0.150

Lag2	0.065	[-0.263,0.393]	(0.167)	0.697	0.278	[-0.114,0.671]	(0.200)	0.165

Note: The association of starting exercise habits is estimated with a subsample of only groups (A) and (B) (n = 16512). In contrast, The association of quitting is estimated with a subsample of only groups (D) and (E) (n = 6060). The Lead variable is a binary variable indicating the ex-ante period of exercise habit change. The Lag variable is a binary variable indicating the ex-post period of exercise habit change. Lag0 indicates the period immediately after the change in exercise habits. Standard errors are clustered by individuals. Statistical significance was set as follows: * p < .05, ** p < .01, *** p< .001. **Abbreviations**: 95%CI, 95% confidence interval; SE, standard error;

Table S3. Association dynamics of changes in exercise habits on work engagement and presenteeism for gender subsamples

**For male sample**		Association of starting exercise (N=10 896)				Association of quitting exercise (N=4892)			
Outcome	Relative wave	Coefficient	[95%CI]	(SE)	p-value	Coefficient	[95%CI]	(SE)	p-value
Work engagement	Lead3	-0.059	[-0.207,0.089]	(0.075)	0.435	-0.099	[-0.353,0.155]	(0.129)	0.444
	Lead2	-0.014	[-0.147,0.119]	(0.068)	0.833	-0.012	[-0.209,0.185]	(0.100)	0.908
	Lead1	Base	Base	Base	Base	Base	Base	Base	Base
	Lag0	0.152**	[0.051,0.253]	(0.052)	0.003	-0.258***	[-0.409,-0.106]	(0.077)	0.001
	Lag1	0.190*	[0.045,0.336]	(0.074)	0.010	-0.201*	[-0.378,-0.025]	(0.090)	0.026
	Lag2	0.290**	[0.115,0.465]	(0.089)	0.001	-0.167	[-0.406,0.073]	(0.122)	0.173
Presenteeism	Lead3	-0.163	[-0.496,0.170]	(0.170)	0.337	-0.028	[-0.630,0.573]	(0.307)	0.927
	Lead2	0.110	[-0.141,0.361]	(0.128)	0.390	-0.004	[-0.395,0.387]	(0.199)	0.985
	Lead1	Base	Base	Base	Base	Base	Base	Base	Base
	Lag0	0.115	[-0.116,0.346]	(0.118)	0.329	0.270	[-0.003,0.543]	(0.139)	0.052
	Lag1	0.088	[-0.201,0.377]	(0.148)	0.551	0.246	[-0.101,0.593]	(0.177)	0.164
	Lag2	0.224	[-0.160,0.608]	(0.196)	0.253	0.390	[-0.073,0.852]	(0.236)	0.099
**For female sample**		Association of starting exercise (N=5616)				Association of quitting exercise (N=1168)			
Outcome	Relative wave	Coefficient	[95%CI]	(SE)	p-value	Coefficient	[95%CI]	(SE)	p-value
Work engagement	Lead3	0.131	[-0.121,0.383]	(0.128)	0.307	-0.353	[-0.815,0.109]	(0.235)	0.134
	Lead2	0.037	[-0.202,0.276]	(0.122)	0.761	-0.110	[-0.413,0.194]	(0.154)	0.477
	Lead1	Base	Base	Base	Base	Base	Base	Base	Base
	Lag0	0.103	[-0.074,0.281]	(0.090)	0.254	-0.086	[-0.362,0.190]	(0.140)	0.539
	Lag1	0.241*	[0.016,0.466]	(0.115)	0.035	-0.138	[-0.483,0.208]	(0.176)	0.433
	Lag2	0.411**	[0.121,0.700]	(0.148)	0.006	0.015	[-0.458,0.489]	(0.241)	0.950
Presenteeism	Lead3	0.141	[-0.346,0.628]	(0.248)	0.569	0.568	[-0.445,1.580]	(0.514)	0.271
	Lead2	0.046	[-0.329,0.421]	(0.191)	0.810	0.594	[-0.142,1.331]	(0.374)	0.113
	Lead1	Base	Base	Base	Base	Base	Base	Base	Base
	Lag0	-0.248	[-0.610,0.115]	(0.185)	0.181	0.105	[-0.348,0.558]	(0.230)	0.648
	Lag1	-0.372	[-0.892,0.147]	(0.265)	0.160	0.149	[-0.427,0.724]	(0.292)	0.612
	Lag2	-0.378	[-1.000,0.244]	(0.317)	0.233	-0.077	[-0.807,0.653]	(0.371)	0.836

Note: The association of starting exercise habits is estimated with a subsample of only groups (A) and (B) (n = 16 512). In contrast, The association of quitting is estimated with a subsample of only groups (D) and (E) (n = 6060). The Lead variable is a binary variable indicating the ex-ante period of exercise habit change. The Lag variable is a binary variable indicating the ex-post period of exercise habit change. Lag0 indicates the period immediately after the change in exercise habits. Standard errors are clustered by individuals. Statistical significance was set as follows: * p < .05, ** p < .01, *** p< .001. **Abbreviations**: 95%CI, 95% confidence interval; SE, standard error;

Table S4. Association dynamics of changes in exercise habits on work engagement and presenteeism with robust estimation from heterogeneity in the timing of exercise habits changes

		Association of starting exercise (N=16 512)				Association of quitting exercise (N=6060)			
Outcome	Relative wave	Coefficient	[95%CI]	(SE)	p-value	Coefficient	[95%CI]	(SE)	p-value
Work engagement	Lead3	-0.024	[-0.162,0.114]	(0.070)	0.737	-0.238	[-0.493,0.018]	(0.130)	0.069
	Lead2	-0.026	[-0.146,0.094]	(0.061)	0.672	-0.017	[-0.191,0.156]	(0.088)	0.845
	Lead1	Base	Base	Base	Base	Base	Base	Base	Base
	Lag0	0.137**	[0.049,0.225]	(0.045)	0.002	-0.224**	[-0.357,-0.092]	(0.068)	0.001
	Lag1	0.221**	[0.086,0.356]	(0.069)	0.001	-0.151	[-0.316,0.015]	(0.084)	0.074
	Lag2	0.356***	[0.190,0.523]	(0.085)	<0.001	-0.142	[-0.375,0.092]	(0.119)	0.235
Presenteeism	Lead3	-0.047	[-0.337,0.244]	(0.148)	0.752	0.281	[-0.281,0.844]	(0.287)	0.327
	Lead2	0.120	[-0.087,0.328]	(0.106)	0.256	0.249	[-0.109,0.607]	(0.183)	0.173
	Lead1	Base	Base	Base	Base	Base	Base	Base	Base
	Lag0	0.021	[-0.175,0.217]	(0.100)	0.833	0.227	[-0.006,0.461]	(0.119)	0.057
	Lag1	-0.041	[-0.314,0.233]	(0.140)	0.771	0.149	[-0.159,0.458]	(0.157)	0.342
	Lag2	0.027	[-0.339,0.392]	(0.187)	0.885	0.160	[-0.263,0.583]	(0.216)	0.459

Note: The association of starting exercise habits is estimated with a subsample of only groups (A) and (B) (n = 16 512). In contrast, The association of quitting is estimated with a subsample of only groups (D) and (E) (n = 6060). The Lead variable is a binary variable indicating the ex-ante period of exercise habit change. The Lag variable is a binary variable indicating the ex-post period of exercise habit change. Lag0 indicates the period immediately after the change in exercise habits. Standard errors are clustered by individuals. Statistical significance was set as follows: * p < .05, ** p < .01, *** p< .001. **Abbreviations**: 95%CI, 95% confidence interval; SE, standard error;

Table S5. Association dynamics of changes in exercise habits on work engagement and presenteeism with robust estimation controlling life events

		Association of starting exercise (N=16 512)				Association of quitting exercise (N=6060)			
Outcome	Relative wave	Coefficient	[95%CI]	(SE)	p-value	Coefficient	[95%CI]	(SE)	p-value
Work engagement	Lead3	-0.002	[-0.130,0.126]	(0.065)	0.978	-0.154	[-0.378,0.071]	(0.114)	0.179
	Lead2	0.000	[-0.116,0.117]	(0.059)	0.996	-0.033	[-0.200,0.134]	(0.085)	0.699
	Lead1	Base	Base	Base	Base	Base	Base	Base	Base
	Lag0	0.138**	[0.050,0.226]	(0.045)	0.002	-0.220**	[-0.354,-0.087]	(0.068)	0.001
	Lag1	0.205**	[0.082,0.327]	(0.063)	0.001	-0.193*	[-0.349,-0.036]	(0.080)	0.016
	Lag2	0.322***	[0.172,0.472]	(0.077)	0.000	-0.126	[-0.340,0.089]	(0.109)	0.250
Presenteeism	Lead3	-0.075	[-0.351,0.201]	(0.141)	0.593	0.077	[-0.447,0.602]	(0.267)	0.772
	Lead2	0.092	[-0.117,0.301]	(0.107)	0.390	0.129	[-0.218,0.477]	(0.177)	0.465
	Lead1	Base	Base	Base	Base	Base	Base	Base	Base
	Lag0	0.020	[-0.176,0.215]	(0.100)	0.844	0.218	[-0.018,0.454]	(0.120)	0.070
	Lag1	-0.028	[-0.281,0.224]	(0.129)	0.826	0.226	[-0.071,0.524]	(0.152)	0.136
	Lag2	0.064	[-0.263,0.391]	(0.167)	0.701	0.281	[-0.113,0.675]	(0.201)	0.161

Note: The association of starting exercise habits is estimated with a subsample of only groups (A) and (B) (n = 16 512). In contrast, The association of quitting is estimated with a subsample of only groups (D) and (E) (n = 6060). The Lead variable is a binary variable indicating the ex-ante period of exercise habit change. The Lag variable is a binary variable indicating the ex-post period of exercise habit change. Lag0 indicates the period immediately after the change in exercise habits. This estimation controls weekly working hours and dummy variables for marital status, presence of children, and life events in the past 6 months (marriage, birth of a child, child advancement in school level, divorce, spouse changing job, spouse quitting job, severe family illness, family requirement for caregiving). Standard errors are clustered by individuals. Statistical significance was set as follows: * $p < .05$, ** $p < .01$, *** $p < .001$. **Abbreviations**: 95%CI, 95% confidence interval; SE, standard error.

Table S6. Association dynamics of quitting exercise habits on working hours for gender subsamples

**For male sample (N=4892)**		Association of quitting exercise			
Outcome	Relative wave	Coefficient	[95%CI]	(SE)	p-value
Working hours	Lead3	-1.215	[-5.820,3.390]	(2.347)	0.605
	Lead2	-0.839	[-4.135,2.457]	(1.680)	0.618
	Lead1	Base	Base	Base	Base
	Lag0	-0.148	[-2.619,2.322]	(1.259)	0.906
	Lag1	-1.274	[-4.297,1.748]	(1.541)	0.408
	Lag2	-0.317	[-3.966,3.333]	(1.860)	0.865
**For female sample (N=1168)**					
Working hours	Lead3	1.054	[-7.722,9.831]	(4.459)	0.813
	Lead2	2.237	[-2.968,7.441]	(2.644)	0.398
	Lead1	Base	Base	Base	Base
	Lag0	4.284	[-0.242,8.810]	(2.300)	0.063
	Lag1	-0.892	[-7.313,5.529]	(3.263)	0.785
	Lag2	0.322	[-6.743,7.387]	(3.590)	0.929

Note: The association of quitting is estimated with a subsample of only groups (D) and (E) (n = 6060). The Lead variable is a binary variable indicating the ex-ante period of exercise habit change. The Lag variable is a binary variable indicating the ex-post period of exercise habit change. Lag0 indicates the period immediately after the change in exercise habits. Standard errors are clustered by individuals. Statistical significance was set as follows: * p < .05, ** p < .01, *** p< .001. **Abbreviations**: 95%CI, 95% confidence interval; SE, standard error.

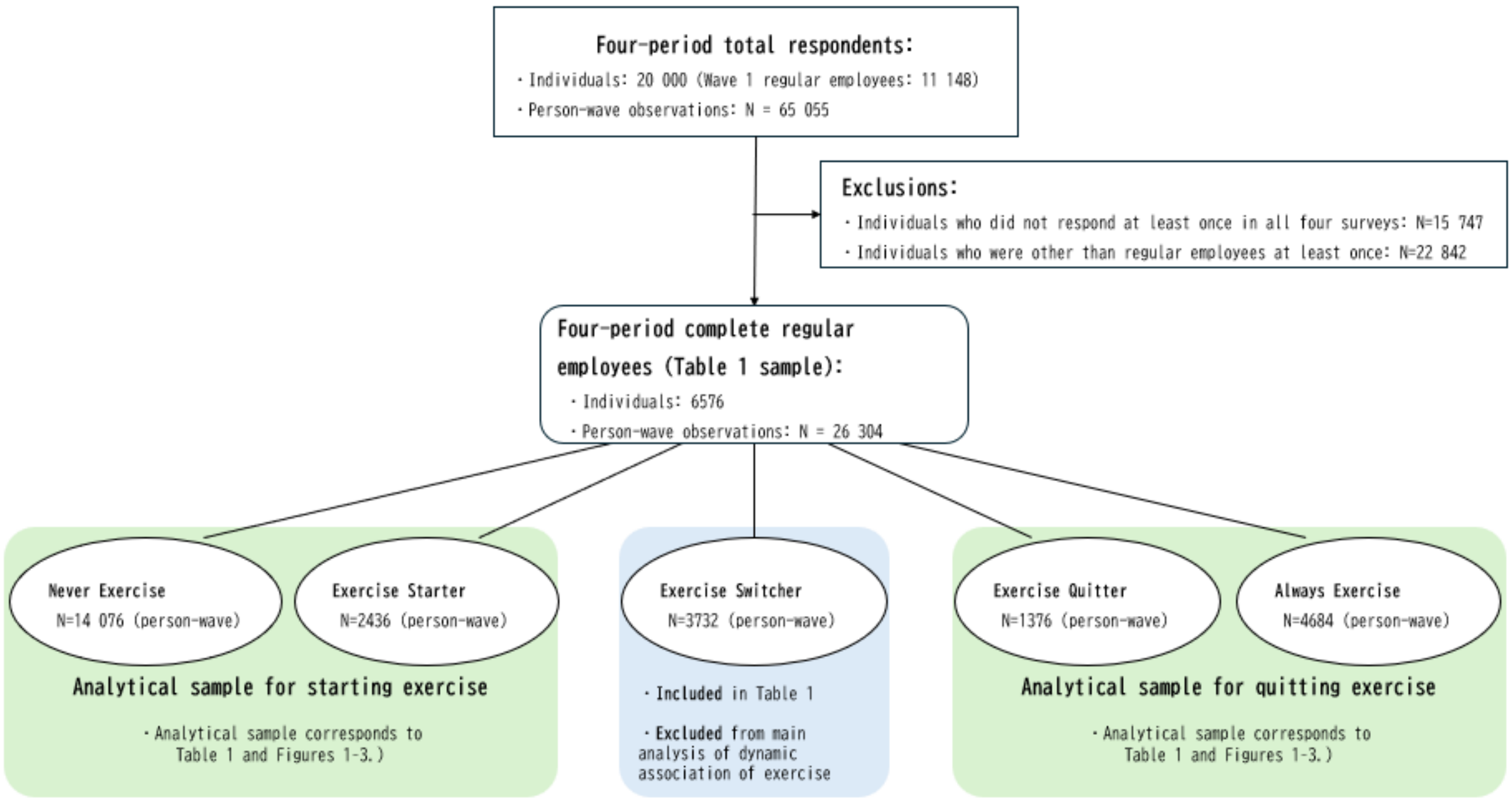

Figure S1. Flowchart of the participant and sample selection process

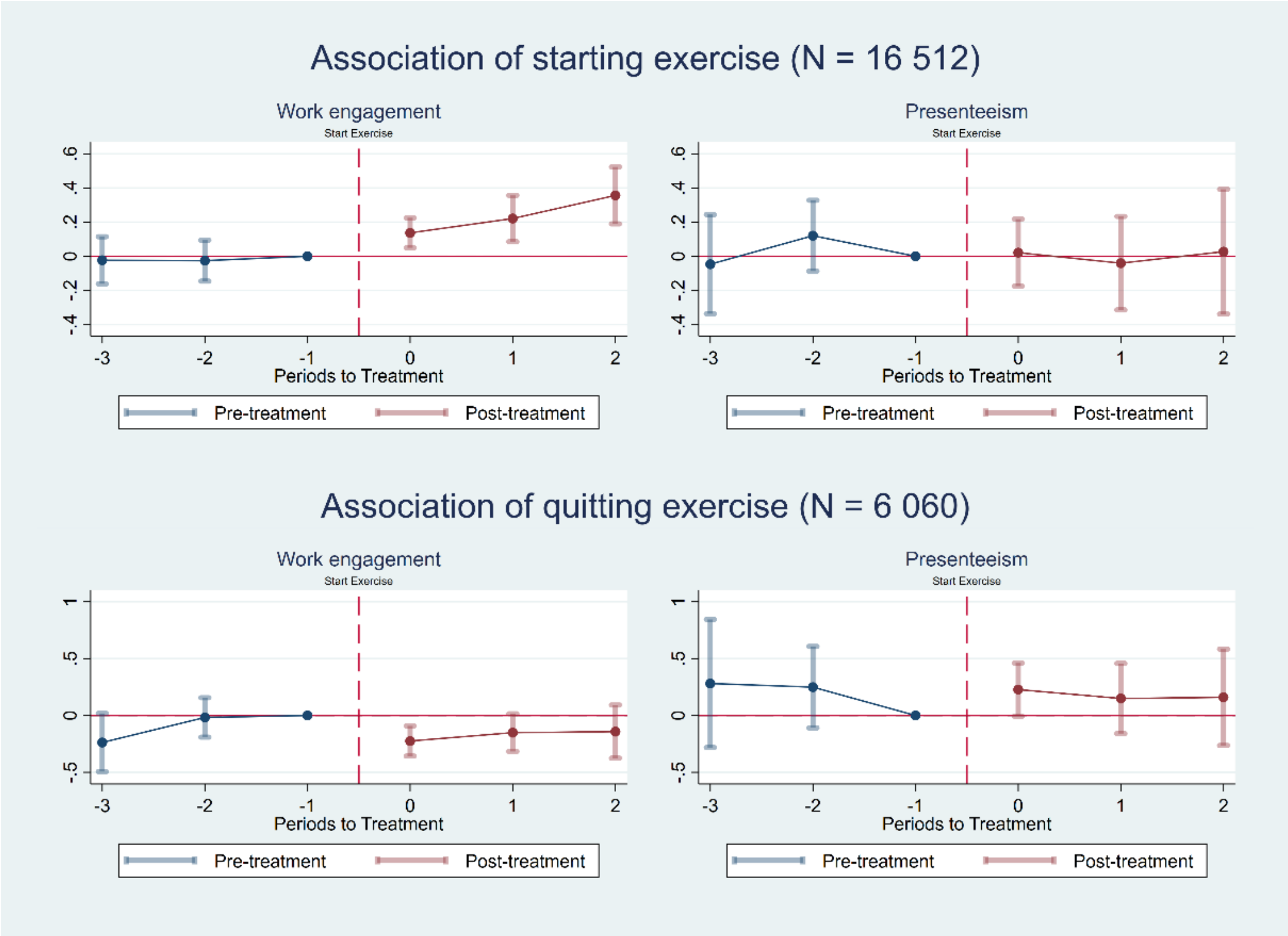

Figure S2. Association dynamics of changes in exercise habits on work engagement and presenteeism with robust estimation from heterogeneity in the timing of exercise habits changes.

Note: The upper part of the figure estimates the starting exercise habit effect using a subsample of only groups (A) and (B) (n = 16 512). In contrast, the lower part estimates the quitting exercise habits effect using a subsample of only groups (D) and (E) (n = 6 060). The x-axis shows the relative wave from the survey wave where exercise habits changed. The area to the left of the vertical dashed line indicates the effect before the change in exercise habits, while the area to the right indicates the effect after the change in exercise habits. Table S4 corresponds with this figure.

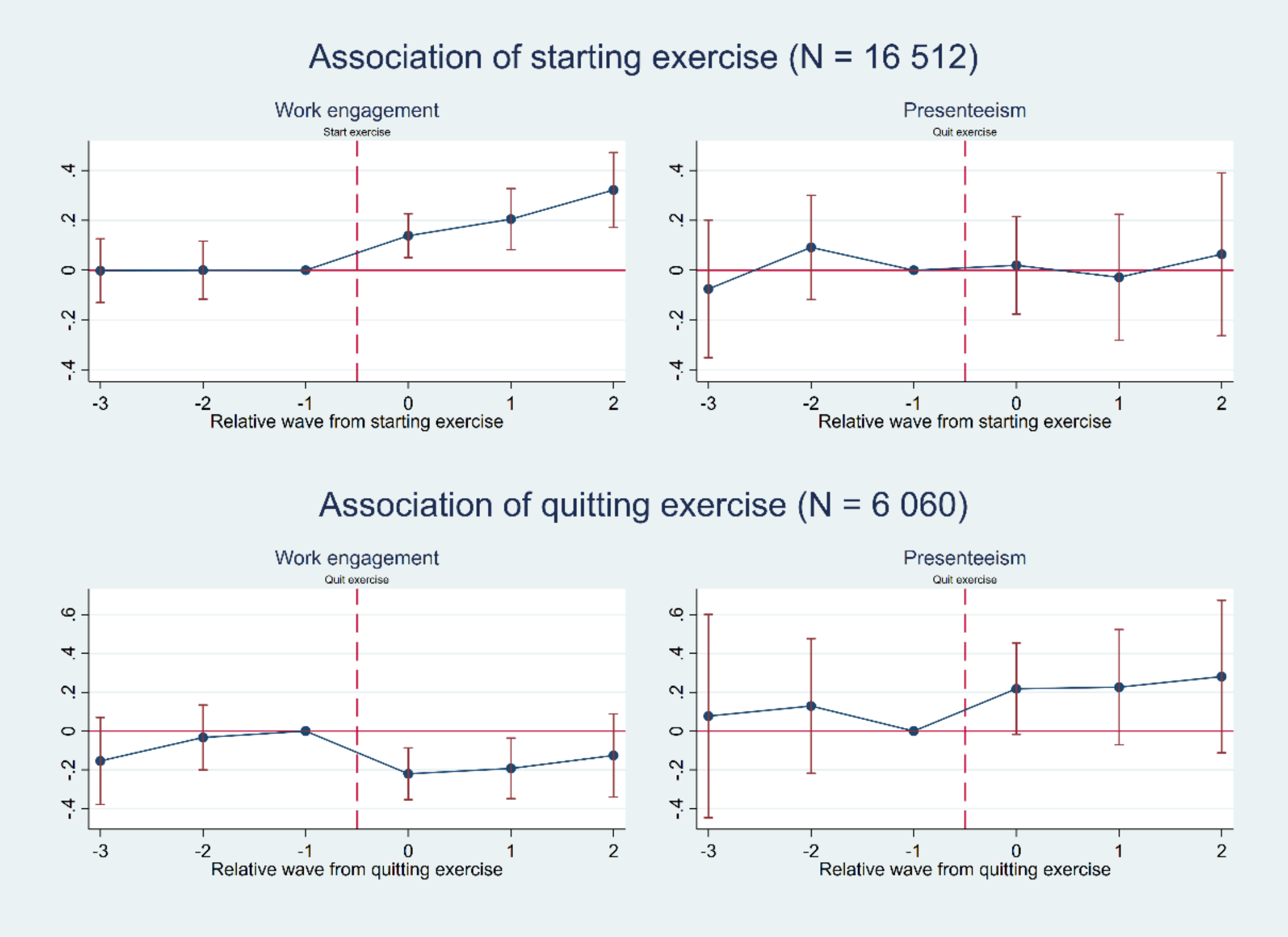

Figure S3. Association dynamics of changes in exercise habits on work engagement and presenteeism with robust estimation controlling life events.

Note: The upper part of the figure estimates the starting exercise habit effect using a subsample of only groups (A) and (B) (n = 16 512). In contrast, the lower part estimates the quitting exercise habits effect using a subsample of only groups (D) and (E) (n = 6 060). The x-axis shows the relative wave from the survey wave where exercise habits changed. The area to the left of the vertical dashed line indicates the effect before the change in exercise habits, while the area to the right indicates the effect after the change in exercise habits. Table S5 corresponds with this figure. This estimation controls weekly working hours and dummy variables for marital status, presence of children, and life events in the past 6 months (marriage, birth of a child, child advancement in school level, divorce, spouse changing job, spouse quitting job, severe family illness, family requirement for caregiving).

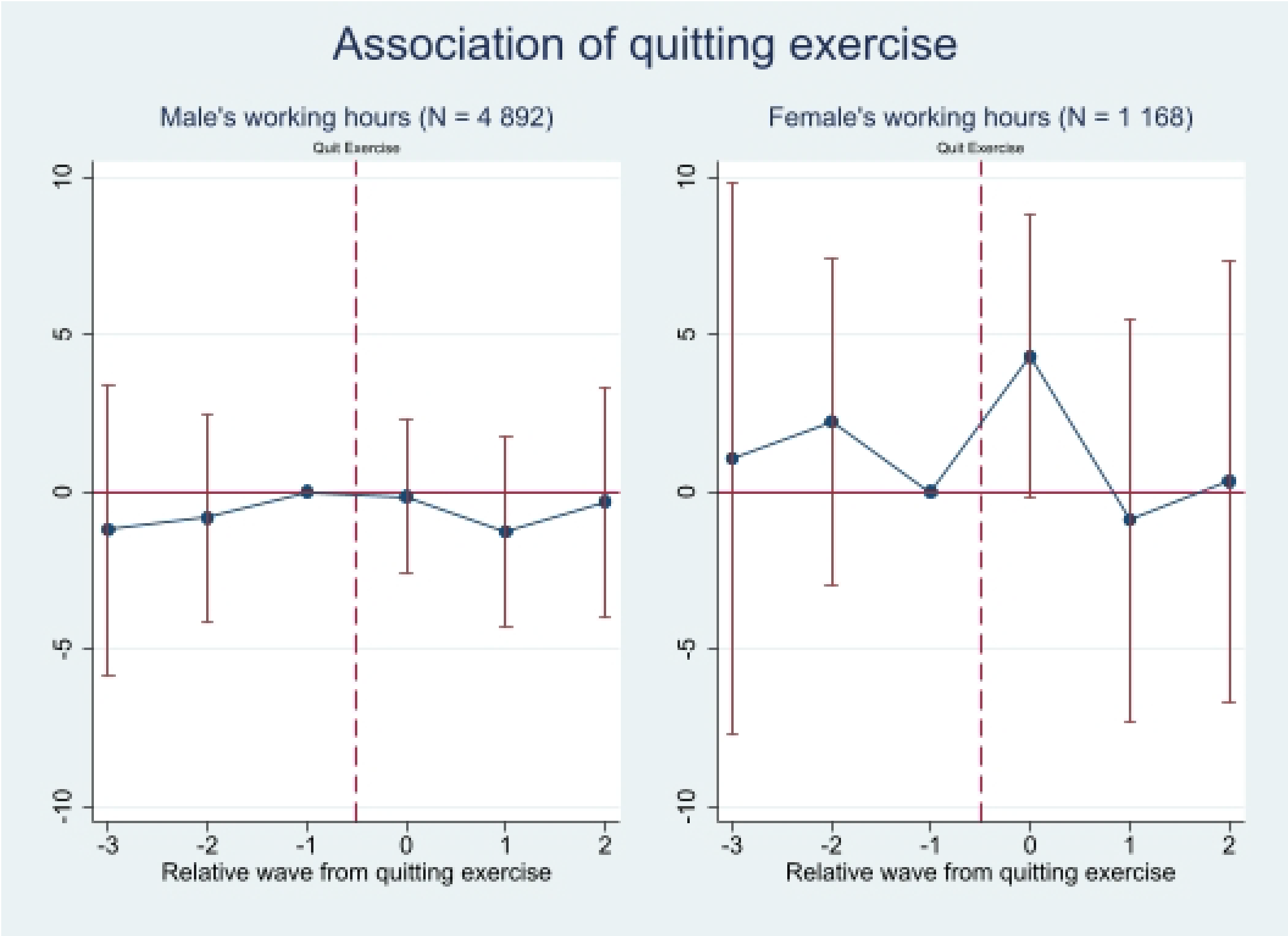

Figure S4. Association dynamics of quitting exercise habits on working hours for gender subsamples. Note: The figure estimates The association of quitting exercise habits on working hours using a subsample of only groups (D) and (E) in each gender subsample (n = 6 060). The x-axis shows the relative wave from the survey wave where exercise habits changed. The area to the left of the vertical dashed line indicates the effect before the change in exercise habits, while the area to the right indicates the effect after the change in exercise habits. Table S6 corresponds with this figure.

Appendix A: Details of event-study analysis (main estimation of this study)

As mentioned in the main text, we conducted event-study estimation based on fixed-effect models. The standard event-study specification is as follows:

$$y_{it} = \sum_{p=-3}^{-2} \beta_p \, Lead_{it}^{p} + \sum_{k=0}^{2} \gamma_k \, Lag_{it}^{k} + \eta_i + \eta_t + u_{it}$$

The subscript i represents the individuals, t represents the survey wave, and p, k indicates the relative wave from right after the change in exercise habits. $Lead_{it}^{p}$ and Lag_{it}^{k} are dummies, and they equal 1 if the survey period is p or k period away from the wave where individuals change their exercise habits. Here, the coefficient γ_k captures the impact of the change in exercise habits in the relative period k. This estimation allows us to check the association dynamics. To interpret the estimated coefficient as close results to causal relationship, the parallel

trends assumption should be satisfied. If β_p are not statistically significant, we can consider the parallel trends assumption as supported (non-violated), indicating that there was no difference in the outcome trends between the treatment and control groups before the habit changes. We conducted above estimation method by using "hdfe" packages in STATA.

The two estimation methods above may produce biased results when treatment timing varies or when the magnitude of coefficients changes over time (36). As robustness check in Figure S2, we relied on Wooldridge (2021 & 2023)'s modified estimation method (42,43). We followed the modified method using "jwdid" (41) in STATA.
